# Impact of Sublethal Levels of Environmental Pollutants Found in Sewage Sludge on a Novel *Caenorhabditis elegans* Model Biosensor

**DOI:** 10.1371/journal.pone.0046503

**Published:** 2012-10-03

**Authors:** Debbie McLaggan, Maria R. Amezaga, Eleni Petra, Andrew Frost, Elizabeth I. Duff, Stewart M. Rhind, Paul A. Fowler, L. Anne Glover, Cristina Lagido

**Affiliations:** 1 Institute of Medical Sciences, University of Aberdeen, Aberdeen, Scotland, United Kingdom; 2 The James Hutton Institute, Aberdeen, Scotland, United Kingdom; 3 Biomathematics and Statistics Scotland, Aberdeen, Scotland, United Kingdom; The University of Chicago, United States of America

## Abstract

A transgenic strain of the model nematode *Caenorhabditis elegans* in which bioluminescence reports on relative, whole-organism ATP levels was used to test an environmentally-relevant mixture of pollutants extracted from processed sewage sludge. Changes in bioluminescence, following exposure to sewage sludge extract, were used to assess relative ATP levels and overall metabolic health. Reproductive function and longevity were also monitored. A short (up to 8 h) sublethal exposure of L4 larval stage worms to sewage sludge extract had a concentration-dependent, detrimental effect on energy status, with bioluminescence decreasing to 50–60% of the solvent control (1% DMSO). Following longer exposure (22–24 h), the energy status of the nematodes showed recovery as assessed by bioluminescence. Continuous exposure to sewage sludge extract from the L4 stage resulted in a shorter median lifespan relative to that of solvent or medium control animals, but only in the presence of 400–600 µM 5-fluoro-2′-deoxyuridine (FUdR), which was incorporated to inhibit reproduction. This indicated that FUdR increased lifespan, and that the effect was counteracted by SSE. Exposure to sewage sludge extract from the L1 stage led to slower growth and a delayed onset of egg laying. When L1 exposed nematodes reached the reproductive stage, no effect on egg laying rate or egg number in the uterus was observed. DMSO itself (1%) had a significant inhibitory effect on growth and development of *C. elegans* exposed from the L1 stage and on reproduction when exposed from the L4 stage. Results demonstrate subtle adverse effects on *C. elegans* of a complex mixture of environmental pollutants that are present, individually, in very low concentrations and indicate that our biosensor of energy status is a novel, sensitive, rapid, quantitative, whole-organism test system which is suitable for high throughput risk assessment of complex pollutant mixtures.

## Introduction

Many anthropogenic chemicals are present in the environment at concentrations below those considered likely to trigger health concerns. However, when combined in complex mixtures, these chemicals may exert adverse effects on physiology and health [1], [2]. One source of an environmentally-relevant mixture of pollutants is processed sewage sludge, a by-product of waste water treatment that has long been applied to agricultural and brown-field land as a fertiliser or soil remediation treatment [3]. The advantages of using sewage sludge to improve soil quality are that it is cheap compared to inorganic fertiliser, contains suitable levels of nutrients of agricultural value such as nitrogen and phosphorus, and serves as a convenient means of disposal of sewage sludge waste. The major disadvantage is that it also contains a cocktail of heavy metal and persistent organic pollutants (POPs). The latter, like heavy metals, are resistant to environmental degradation and can therefore accumulate in animal tissues. Each component is present individually at low concentrations, relative to those known to be biologically harmful. These pollutants include chemicals used in the manufacture of plastics (phthalates), electrical equipment (polychlorinated biphenyls (PCBs)), and flame retardants (polybrominated diphenyl ethers (PBDEs)). Additional pollutants present in SSE include polycyclic aromatic hydrocarbons (PAHs), which are derived from incomplete combustion of hydrocarbon fuels [4].

Mammalian exposure to persistent pollutants commonly occurs through air and water [5], but since they are not easily degraded, human and animal exposure can be elevated as a result of biomagnification in the higher levels of food chains. Therefore, carnivores are particularly susceptible to high rates of tissue accumulation of these chemicals [6]. However, effects also occur in animals lower down the food chain; e.g. *in utero* exposure to these environmental pollutants, many of which are endocrine disrupting compounds (EDCs), has detrimental effects on reproductive, neuroendocrine and gonadal functions in offspring of sheep pastured on sewage-sludge fertilised fields [7]–[10], persisting into adulthood [11]. While experiments involving large animals provide critical insight into the effects of exposure, they are expensive and time-consuming studies and thus inappropriate for routine screening of complex mixtures of chemicals. Other methods better suited to high throughput are therefore required for initial and/or mechanistic screening of pollutant mixtures.

We have developed a new non-mammalian tool to facilitate the assessment of effects of environmental toxic stress [12]. *Caenorhabditis elegans* is a well-characterised model organism used to study many mammalian physiological processes, including stress response mechanisms [13]. Although an endocrine system *per se* has not been identified in *C. elegans*, it shares with higher organisms many processes that are regulated via conserved hormonal/receptor pathways, such as the involvement of the target of the rapamycin (TOR) signalling pathway and of insulin/IGF signalling in stress responses and longevity [13]. Furthermore, endocrine effects of xenobiotics that match typical symptoms of endocrine disruption have been described for *C. elegans* and other nematodes at the molecular, organismal and community levels (reviewed in [14]). We have generated luminescent ATP sensor *C. elegans* strains through whole-organism constitutive expression of the firefly luciferase gene. These transgenic worms exhibit reduced light output in response to knockdown of mitochondrial respiratory genes or treatment with xenobiotics [15] and so provide a real-time read-out of metabolic status [16]. We validated bioluminescence as a toxicological endpoint by demonstrating that the decline in bioluminescence upon exposure to the model toxicant cadmium (Cd) was a rapid indicator of toxicity, which showed comparable sensitivity to other conventional sublethal endpoints, such as reproduction and development [12]. The physiological impact of a mixture of pollutants, individually present at low environmental concentrations, is however, more difficult to determine, and the aim of this study was to assess the value of our biosensor *C. elegans* in this regard. We show that a sewage sludge extract exerts sublethal effects on this model nematode and that the determination of relative ATP levels by bioluminescence is a powerful means of rapid sublethal toxicity screening for complex mixtures of environmental chemicals.

## Results

### Chemical Composition of Sewage Sludge Extract (SSE)

The concentrations of representative xenobiotics from four major pollutant classes (i.e. PAHs, PBDEs, PCB and metals) contained in SSE are shown in Table 1.

**Table 1:** Representative organic pollutants and potentially toxic metals contained in 1% sewage sludge extract.

Compound/metal	µg/l	
Naphthalene	0.500	
Acenaphthalene	0.036	
Acenaphthene	0.092	
Fluorene	0.776	
Phenanthrene	1.190	
Anthracene	0.368	
Fluoranthene	9.480	
Pyrene	3.782	
Benzo[a]anthracene	1.372	
Chrysene	7.032	
Benzo[b]fluoranthene	4.111	
Benzo[k]fluoranthene	1.212	
Benzo[a]pyrene	0.152	
Indeno[1,2,3-cd]pyrene	0.288	
Dibenzo[a,h]anthracene	0.204	
Benzo[ghl]perylene	0.256	
DEHP	0.160	
PBDE 28	0.026	
PBDE 47	1.532	
PBDE 99	2.448	
PBDE 100	0.360	
PBDE 153	0.214	
PBDE 154	0.144	
PBDE 183	0.104	
PCB 28	0.060	
PCB 52	0.024	
PCB 101	0.020	
PCB 118	0.024	
PCB 138	0.030	
PCB 153	0.026	
PCB 180	0.022	
Cr	6	
Cu	14	
Zn	16	

The extract was produced from pellets using DCM and then re-solubilised in DMSO.

### Effect of Exposure to SSE on Metabolic Health Determined by Bioluminescence

**Figure 1:**
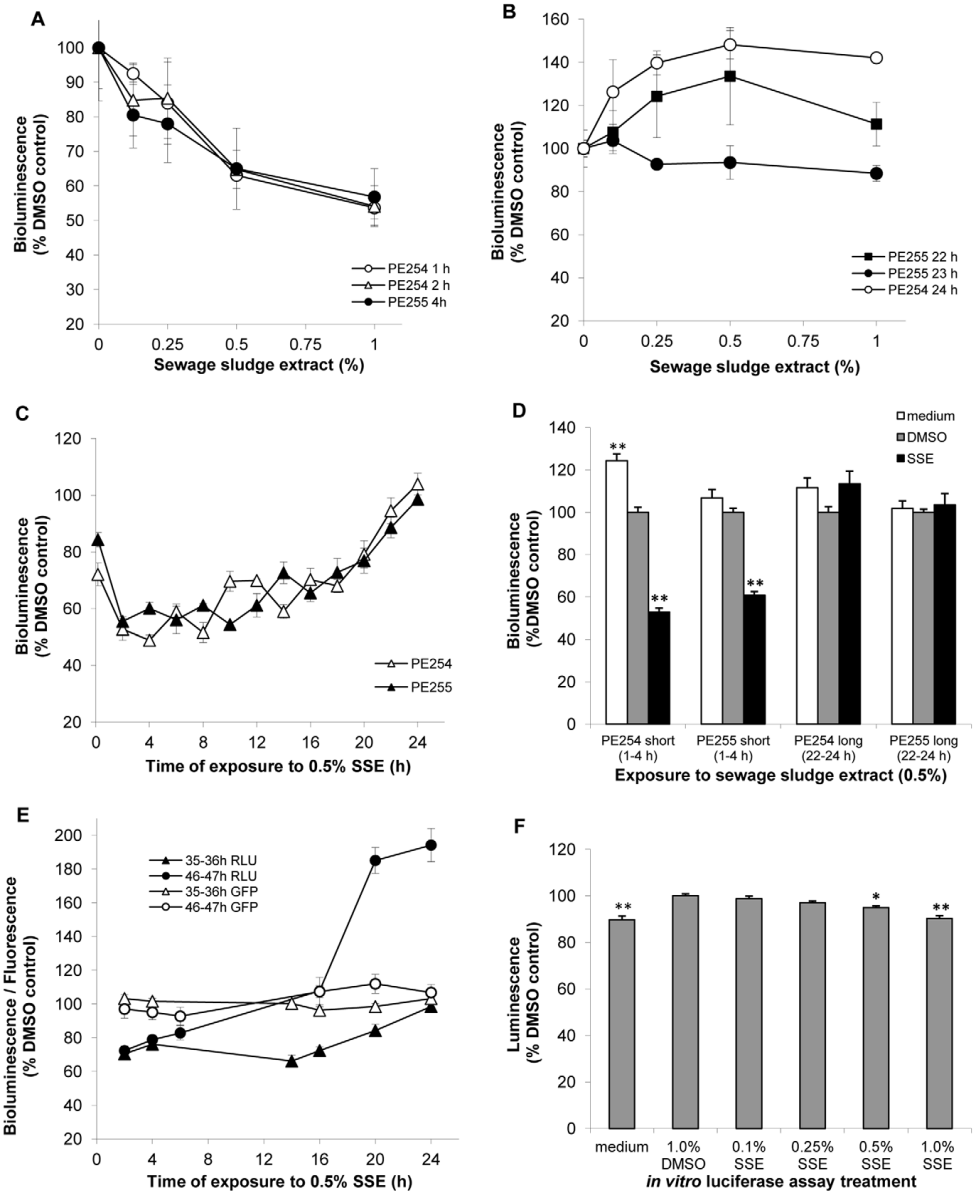
Metabolic health of C. elegans is compromised after short but not long exposure to SSE. Synchronised, L4 stage worms (46–47 h) growing in S-complete medium with OP50 as food source were exposed to SSE or DMSO in 96 well plates. Bioluminescence was expressed as a percentage of the DMSO (1%) solvent control values. (A) Short exposure to SSE (0, 0.1, 0.25, 0.5 and 1.0%): 3 independent experiments carried out at different times shown. Error bars for each experiment depict SEM of technical replicates at each SSE concentration (n = 3). SSE had a significant effect on bioluminescence p<0.001 (no difference between experiments p>0.05). (B) Long exposure to SSE (0, 0.1, 0.25, 0.5 and 1.0%): 3 independent experiments carried out at different times shown. Significant effect of SSE concentration on bioluminescence: closed squares (p = 0.004), open circles (p<0.001); whereas non-significant for closed circles (p>0.05). Error bars depict SEM of technical replicates at each SSE concentration (n = 3). (C) 24 h time course of exposure to 0.5% SSE for PE254 and PE255. Error bars for 0–6 h and 16–24 h depict SEM of 12 technical replicates pooled from 4 independent experiments (8–12 h, 3 technical replicates from 1 experiment; 14 h, 6 technical replicates pooled from 2 independent experiments). (D) Mean bioluminescence values for 0.5% SSE (vehicle 1% DMSO) and respective controls from experiments shown in A–C. Error bars depict SEM of 14–18 technical replicates (pooled from 5–6 independent experiments). ** p<0.001; differences relative to 1% DMSO control. (E) 24 h time course of exposure to 0.5% SSE for ts sterile strain PE328 initiated at the L4 (35–36 h, 25°C) or the adult stage (46–47 h, 25°C). Error bars depict SEM of technical replicates pooled from 3 independent experiments (n = 9 bioluminescence; n = 15 fluorescence). (F) Exposure of purified luciferase protein to the various treatments from A and B. ATP levels were kept constant at 1 mM final concentration. Luminescence was integrated over 10 sec. Error bars equal SEM of technical replicates pooled from 3 independent experiments (n = 12). * p = 0.001; ** p<0.001; differences relative to 1% DMSO control.

Short exposure (1–4 h) of L4 larval stage worms to SSE (0, 0.1, 0.25, 0.5 and 1.0%) in 1% DMSO resulted in a significant reduction in bioluminescence (p<0.001), to 50–60% of solvent control, in an SSE concentration-dependent manner (Fig. 1A); there was no effect on nematode viability. In contrast, prolonged exposure (22–24 h) of L4 *C. elegans* to SSE (0, 0.1, 0.25, 0.5 and 1.0%) did not reduce bioluminescence (Fig. 1B) and on two occasions an increase relative to the DMSO solvent control was observed (p<0.001 in one and p = 0.004 in the other) (Fig. 1B). The magnitude of the increase varied between experiments. To define the time of onset of recovery, a 24 h time course study was carried out for exposure of strains PE254 and PE255 to 0.5% SSE (Fig. 1C). Bioluminescence remained between 50 and 60% of the solvent control for at least 8 h and after 18 h exposure bioluminescence was still only 70% of the solvent control. A subsequent increase in bioluminescence resulted in full recovery by 24 h. To summarize the effects of exposure to SSE, data from 0.5% SSE exposure and respective controls shown in Figures 1A–C were grouped into short (1–4 h) or long (22–24 h) exposures (Fig. 1D). After a short exposure, SSE caused a greater reduction in bioluminescence than DMSO, relative to the medium control (p<0.001). The reduction in bioluminescence caused by the vehicle DMSO, relative to the medium control, was also statistically significant (p = 0.019). Following a long exposure (22–24 h), there was no significant difference in bioluminescence between treatments when all experiments were pooled (p>0.05).

Since the time frame for recovery of bioluminescence coincided with the beginning of the reproductive period, we exposed a temperature sensitive (ts) sterile strain PE328 to SSE (over a 24 h period), in order to establish the potential effects of SSE on somatic cells only (Fig. 1E). Exposure was initiated at two different developmental times: 35–36 h post-hatch (L4 stage reached at 25°C) and 46–47 h post-hatch (adults). When initiated at the L4 stage, SSE exposure for 16 h reduced bioluminescence to 72% of the solvent control, with bioluminescence thereafter increasing to 98% after 24 h; a similar effect seen with PE254 and PE255 (also exposed to SSE at L4 stage) (Fig. 1C). Exposure at the adult stage resulted in a faster recovery, with full recovery achieved by 16 h and bioluminescence of SSE treated nematodes increasing to twice that of DMSO treated worms by 20–24 h exposure (Fig. 1E). As the luciferase protein is fused to GFP, fluorescence was monitored in parallel to bioluminescence in both sets of experiments to measure the levels of enzyme present. No significant changes in fluorescence occurred during the 24 h exposure period when started at the L4 stage (p>0.05). However, longer exposures (16–24 h) of adults led to a small but significant increase in fluorescence in the SSE treatment relative to the DMSO control when compared with short exposures (4–6 h) (p<0.001: except for 24 h vs 4 h p = 0.002; 20 h vs 2 h p = 0.003).

The possibility of any direct effects of the treatments on the luciferase enzyme activity itself was tested using the purified enzyme and constant ATP levels (Fig. 1F). Direct exposure to DMSO led to a small significant increase in enzyme activity compared with the medium (p<0.001). 1% SSE decreased luciferase activity by 10% (p<0.001) and 0.5% SSE reduced it by 5% (p = 0.001). Lower concentrations of SSE had no effect on enzyme activity (p>0.05).

### Effect of Exposure to SSE from L1 and L4 Larval Stage on Rate of Increase in Bioluminescence, Worm Length, Onset of Egg Laying and Progeny Per Adult

**Figure 2:**
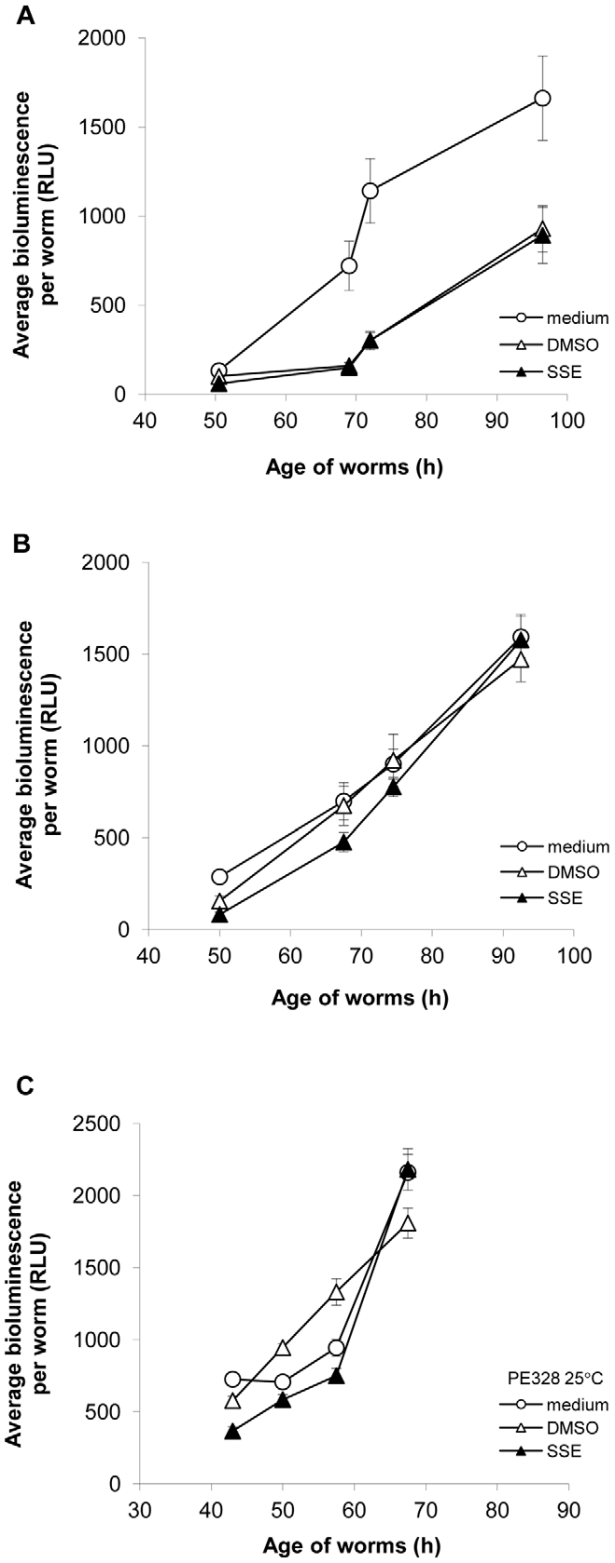
SSE and DMSO exposure at L1 larval stage delayed increase in bioluminescence in PE254 but not ts sterile strain PE328. SSE (1%) or DMSO (1%) was added to: (A) synchronized L1 larval stage, or (B) L4 larval stage (46–47 h) liquid cultures of strain PE254 or (C) synchronized L1 larval stage liquid culture of ts sterile strain PE328. Bioluminescence was determined over comparable developmental times for PE254 (20°C) and PE328 (25°C). Data shown is average bioluminescence per worm from a composite of 4, 2 and 3 independent experiments for (A), (B) and (C) respectively carried out at different times. Error bars depict SEM of technical replicates: A (n = 17); (B) (n = 10); (C) (n = 11).

Exposure to SSE (and DMSO) from the L1 stage resulted in an initial lag in increase in bioluminescence relative to the medium only control (Fig. 2A). After 70 h, the bioluminescence of SSE- and DMSO-exposed nematodes began to rise but the gap in relation to medium only control was not closed by 96 h. In contrast, worms exposed to SSE (or DMSO) from the L4 stage (47 h) showed a rate of increase in bioluminescence comparable to the medium only control from the onset of exposure (Fig. 2B). To remove effects of progeny production, experiments were performed with the bioluminescent ts sterile strain PE328 over a comparable developmental time. These showed no delay in increase in bioluminescence following exposure to SSE (and DMSO) from the L1 stage (Fig. 2C).

**Figure 3:**
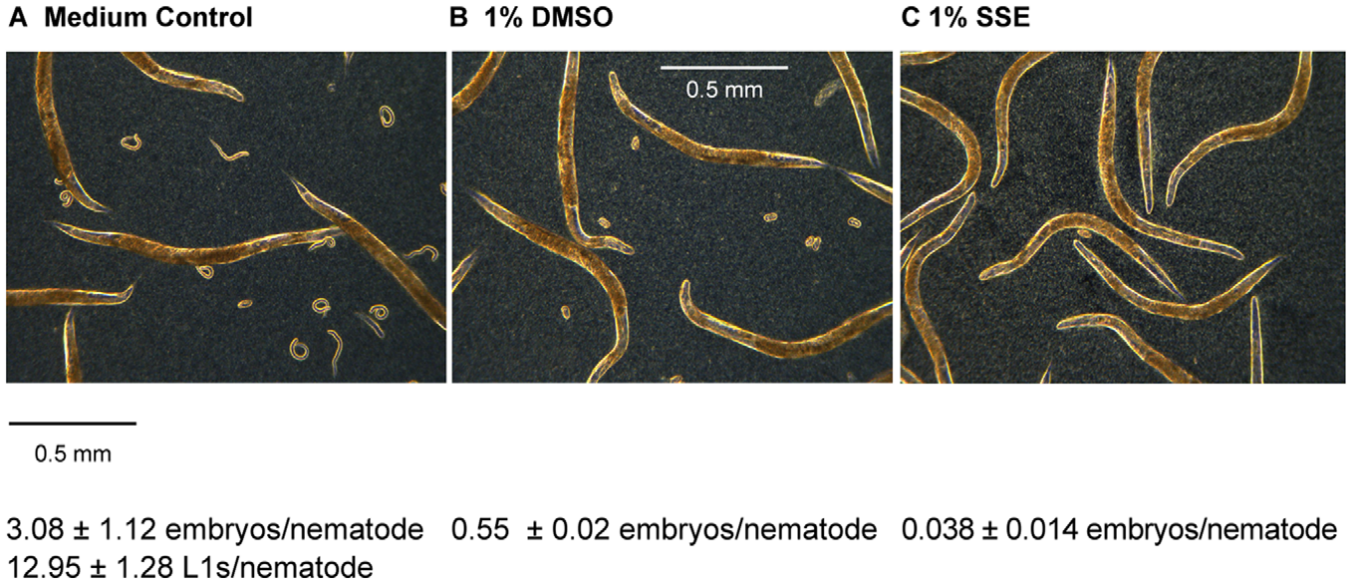
Delayed egg laying at 71.5 h following SSE and DMSO exposure at L1 larval stage. Levamisole anaesthetised worms (1 mM) were photographed with an infinity 2 camera linked to a Leitz Labovert microscope. (A) medium control; (B) DMSO; (C) SSE. Progeny numbers were counted from samples (n = 4) taken at 70.4 h and expressed per nematode. Representative of 3 independent experiments carried out at different times.

This pattern of change in PE254 bioluminescence after exposure from the L1 stage, led to the question of whether growth and development were affected by SSE and DMSO, which was then tested. The length of nematodes exposed to SSE for up to, and including, 74 h was significantly reduced compared to that of worms in DMSO or medium control conditions from the L1 stage (Table 2) (p<0.001). By 96 h exposure, nematode length no longer differed with treatment (p>0.05). DMSO exposure caused an initial delay in growth relative to medium control worms but at 74 h differences were no longer significant. Thus, SSE had an adverse effect on nematode length for a longer period of time than DMSO. The onset of egg laying was also delayed in SSE-exposed nematodes from the L1 stage in relation to DMSO-exposed nematodes: after 70.5 h exposure to SSE from the L1 stage, egg laying remained minimal, whereas nematodes exposed to DMSO alone had eggs/embryos but no hatched progeny; L1 progeny was present only in the controls with no additions (12.95±1.28 L1s/nematode) (Figs. 3 A–C).

**Table 2:** Length (mm) of nematodes exposed to 1% SSE, 1% DMSO or medium alone from the L1 stage.

Exposure (h)	Medium control	1% DMSO	1% SSE
49	0.85±0.01^a*c^ (46)	0.82±0.01^a*b^ (35)	0.76±0.01^b,c^ (35)
69	1.25±0.01^a,c^ (49)	1.19±0.01^a,b^ (40)	1.09±0.01^b,c^ (37)
71.5	1.25±0.01^a*c^ (41)	1.20±0.01^a*b^ (40)	1.12±0.01^b,c^ (41)
74	1.27±0.01^c^ (44)	1.23±0.02^b^ (26)	1.15±0.01^b,c^ (30)
96	1.43±0.01 32)	1.46±0.01 (44)	1.42±0.01 (35)

Average values shown ± SEM (number of nematodes measured). Common superscripts denote significant differences at p<0.001 except for * p = 0.005 (49 h) and p = 0.025 (71.5 h).

**Figure 4:**
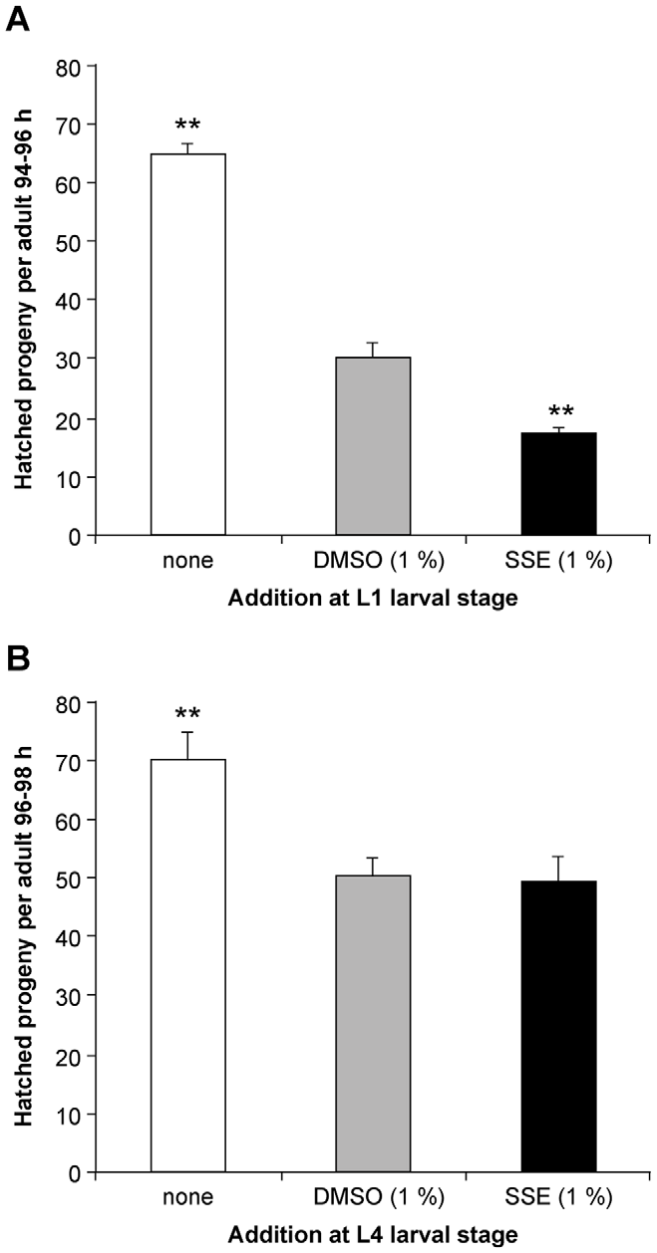
Number of hatched progeny is affected following SSE and DMSO exposure. Hatched progeny from 94–98 h post-hatch adults counted microscopically and expressed per adult. Error bars depict SEM n = 23 representing individual replicate counts from 4 independent experiments. (A) Exposure from L1 larval stage. (B) Exposure from L4 larval stage. ** p<0.001.

The number of hatched progeny per adult (94–98 h post-hatch) following exposure to SSE or DMSO from the L1 stage was significantly reduced relative to the medium control (73% and 53% decline respectively) (p<0.001) (Fig. 4A). There was a significant reduction of 20% for SSE exposure over and above the effect of the DMSO solvent control (p<0.001). When nematodes were exposed from the L4 larval stage, SSE and DMSO exposure reduced hatched progeny per adult significantly to 70.0 and 71.2% of the medium control respectively when determined after 96–98 h post-hatch (p<0.001). However, no significant difference was observed between SSE and DMSO solvent control (Fig. 4B).

### Effect of Exposure to SSE or DMSO from L1 Larval Stage on Egg Laying Rate and Number of Eggs *in utero*

**Figure 5:**
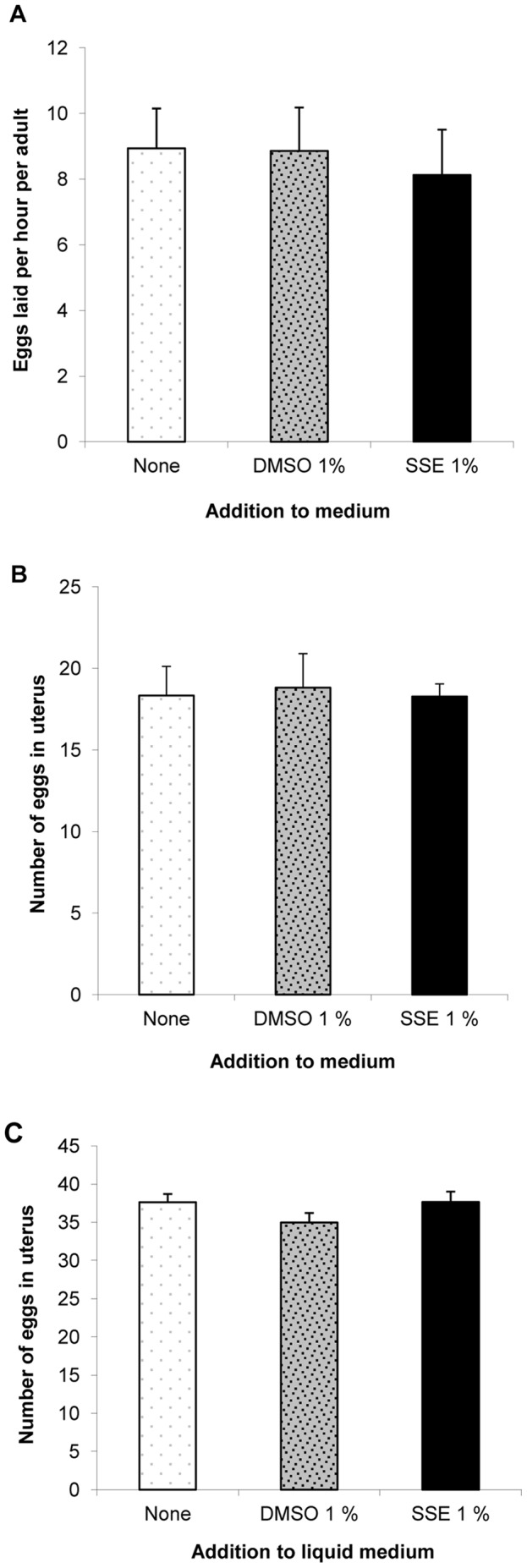
L1 SSE exposure did not affect egg laying rate or *in utero* egg number. (A) Worms grown on NGM plates in the presence or absence of DMSO 1% or SSE 1% were transferred singly to 8 different plates (n = 8): the numbers of eggs laid were determined for the first hour after transfer and then for the second hour and averaged. (B) Adult worms (n = 11) from plates or (C) (n = 23) from liquid culture, grown in the presence or absence of DMSO or SSE, were transferred singly to 10 µl bleach on a microscope slide; after a few minutes the adults dissolved and eggs were counted [43]. (A) represents data from 1 of 2 independent experiments and (B) and (C) represent pooled data from 2 and 3 independent experiments respectively.

SSE and DMSO exposure from the L1 stage on solid growth medium had no effect on the rate of egg laying or on the number of eggs in the uterus of sexually mature nematodes (both similar to the medium control, p>0.05) (Fig. 5A and 5B). Similarly, SSE and DMSO exposure from the L1 stage in liquid medium had no effect on the number of eggs *in utero* (p>0.05) (Fig. 5C). Interestingly, the number of eggs *in utero* for nematodes in liquid culture was higher than for nematodes grown on solid medium.

### Effect of SSE on Median Lifespan

**Figure 6:**
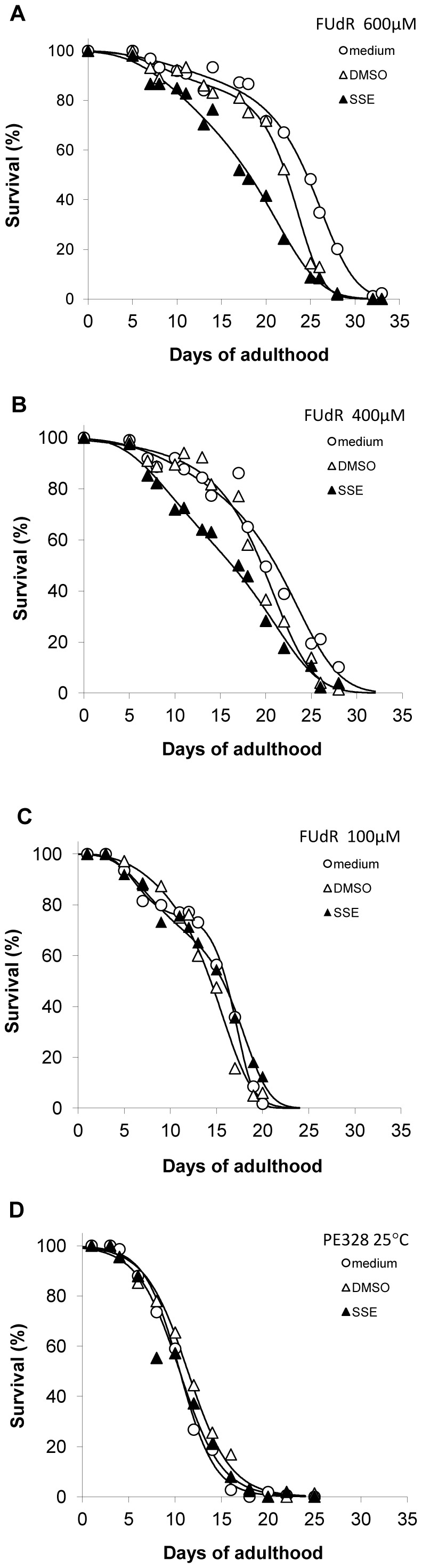
SSE exposure prevents median lifespan increase in the presence of 400 or 600 µM FUdR. FUdR was added to pre-fertile young adults of strain PE254 to prevent progeny development: (A) 600 µM FUdR (B) 400 µM FUdR and (C) 100 µM FUdR. Each culture was grown at 20°C. (D) lifespan monitored in the absence of FUdR, using a temperature sensitive mutant strain PE328 which does not reproduce at 25°C. PE328 was grown at 15°C and shifted to 25°C at the L4 larval stage. 0–600 µM FUdR treatments tested in 3 independent experiments. Approximately 100 worms were scored alive or dead by microscopic observation at each timepoint. Scoring was conducted without knowledge of treatment groups. Fitted curves were derived from cubic (A–C) or linear (D) logistic regression models, using a binomial distribution and logit link function (see Data Analysis section in Methods; Median lifespan estimates are shown in Table 3).

The effect of SSE on lifespan under four different experimental conditions relating to the concentration of FUdR is shown in Figure 6 and the respective median lifespan values are provided in Table 3 (all concentrations of FUdR, including 100 µM, fully blocked progeny production). In the presence of 400 or 600 µM FUdR (Fig. 6B and 6A, respectively) there was a highly significant effect of treatment (SSE, DMSO control, medium control) (p<0.001); nematodes exposed to SSE displayed a shorter median lifespan than the respective DMSO and medium only controls. At 400 µM FUdR, the median lifespan of SSE exposed worms was 16.3 days, more than 3 days shorter than for DMSO exposed nematodes (19.6 days) and nearly 5 days shorter than respective medium only controls (21.1 days). At 600 µM FUdR, the median lifespan of SSE exposed nematodes was 18.1 days, considerably shorter than for DMSO exposed worms (22.3 days) and medium only controls (24.6 days). This effect was dependent on high concentrations of FUdR being present as there was no overall effect of treatment in the presence of 100 µM FUdR (Fig. 6C) (p>0.05). At 100 µM FUdR median lifespan under the different treatments were more similar (15.7 days for SSE, 14.4 for DMSO and 16.1 for medium controls). In addition, in the absence of FUdR, no effect of treatment on lifespan of a ts sterile mutant (PE328) was observed (p>0.05; Fig. 6D).

**Table 3:** Median Lifespan estimates for 0 to 600 µM FUdR in the presence of the treatments 1% SSE, 1% DMSO or medium alone.

Experiment	Medium control	1% DMSO	1% SSE	Treatment effect	Interaction with time
600 µM FUdR	24.58	22.27	18.11	p<0.001	p<0.001
	(23.98, 25.20)	(21.77, 22.79)	(17.32, 18.99)		
400 µM FUdR	21.12	19.59	16.28	p<0.001	p = 0.049
	(20.29, 21.88)	(19.04, 20.20)	(15.38, 17.26)		
100 µM FUdR	16.05	14.37	15.68	p>0.05	p<0.001
	(15.35, 16.64)	(13.82, 14.95)	(14.94, 16.33)		
25°C sterile strain*	10.49	11.36	10.48	p>0.05	p>0.05
	(10.16, 10.82)	(10.95, 11.80)	(10.10, 10.88)		

Lower and upper 95% confidence limits are shown in brackets. Confidence limits were derived using bootstrap resampling from binomial distributions.

* no FUdR. Statistical comparisons refer to Table rows.

An effect of higher concentrations of FUdR on extension of median lifespan was also evident (Table 3). Under medium control conditions, median lifespan of nematodes was extended by more than 8 days when the FUdR concentration was increased from 100 to 600 µM. This increase in median lifespan was less pronounced for SSE exposure: median lifespan was extended by less than 2.5 days when the FUdR concentration was increased from 100 to 600 µM (7.9 days extension for DMSO control). This effect of FUdR can also be seen as a shift to the right in the fitted survival curves in Figure 6.

## Discussion

This study examined the effects on the nematode *C. elegans* of exposure to a complex mixture of environmental chemicals, individually present at low concentrations. The mixture used (SSE), included a wide range of the environmentally-ubiquitous, synthetic, organic compounds contained in sewage sludge fertiliser and, in this respect, is representative of ‘real world’ exposures. Furthermore, the concentrations of each of the chemicals in the nematode culture were of a similar order of magnitude to those reported in the tissues of mammals exhibiting physiological disruptions [6], [10]. However, the contribution of heavy metals in the extract was relatively low (due to the extraction method) compared with sludge fertiliser *per se* [17]. It should be noted that these chemicals represent only a small fraction of the total environmental pollutants that are likely to be present in the extract and so can only be indicative of the environmental burden.

Four main significant effects of SSE versus DMSO solvent control on *C. elegans* physiology were identified: (i) reduced bioluminescence following exposures (8 h or less) at L4 stage indicated lowered ATP levels and compromised metabolic health; conversely (ii) recovery following longer exposures; (iii) slower increase in worm length, later onset of egg laying and reduced brood size at 94–98 h post-hatch following exposure from the L1 stage; and (iv) shorter mean lifespan relative to controls in the presence of high concentrations of FUdR when exposed from L4 stage. Our results have also revealed a vehicle effect on growth and development when 1% DMSO was added to nematodes at the L1 stage and a significant effect on reproduction when added at the L4 stage.

Microbial bioluminescence assays reporting on metabolic perturbation following environmental insults have been used for some time in ecotoxicology [18]–[20]. Previously, we extended this approach to a simple multicellular animal, *C. elegans*, by transgenic expression of firefly luciferase and shown that exposure to single stresses such as heavy metals, 3,5-dichlorophenol, sodium azide or heat, resulted in reduced relative ATP levels [12], [15], [16]. In the present study, we have shown that the environmentally-relevant combination of chemicals present in SSE had a negative impact on transgenic *C. elegans* bioluminescence after exposures at least up to 8 h, which was not associated with lethality or changes in the expression of the luciferase enzyme *in vivo* or activity *in vitro*. Therefore, this is indicative of reduced energetic status of nematodes upon exposure to the chemicals in SSE as previously described for single compounds [12], [15], [16]. Our results are broadly in agreement with another study in which urban sludge containing a range of PAHs, PCBs and metals was shown to inhibit the bioluminescence of the bacterium *Vibrio fisheri,* after 15–30 min exposures [21]. The impact of sludge extracts on bioluminescence is likely to result from both direct metabolic inhibition and from the activation of energetically costly stress responsive mechanisms.

During the course of long exposures to SSE, initiated at the L4 larval stage, the metabolic status of *C. elegans* showed a recovery. *C. elegans* has a diverse genetic toolbox available for detoxification of xenobiotics including cytochrome P450 (CYP) enzymes although not CYP1 [22], short-chain dehydrogenases (SDR), UDP-glucuronosyl or glycosyl transferases (UGT) and glutathione S-transferases (GST) [23]. Similarly to mammals *C. elegans* also possesses xenosensing nuclear receptors that may regulate transcription of these detoxifying enzymes [23]. In addition, when the adult sterile strain was exposed to SSE for 20–24 h, bioluminescence showed a two-fold rise, in comparison to controls. The small increase in GFP fluorescence that occurred (indicative of a small increment in luciferase expression levels associated with growth) did not explain the much greater bioluminescence response. Luciferase activity was also not enhanced by the SSE treatment as shown *in vitro*. This response at 67 h post-hatch (25°C) was therefore consistent with increased ATP levels. This may result from the activity of (over)compensatory energetic mechanisms involving, for example, mitochondrial mass and/or function, induced in response to the prior SSE mediated drop in energy levels [24]. The two-fold increase was not observed when the sterile strain was exposed to SSE for up to 24 h from the L4 stage (60 h post-hatch at 25°C). We propose that the age post-hatch (rather than the time of onset of exposure) is the critical parameter in the steep increase in ATP levels. This will require further investigation.

SSE exposure delayed growth and development from the L1 stage, an effect which was slightly greater than that of the vehicle control as assessed by length measurements and delay in progeny production. This offers a plausible explanation for reduced numbers of progeny per adult at 96 h post-hatch, relative to the DMSO control. Once reproductive maturity was reached, the rate of egg laying and number of eggs in the uterus of worms exposed to SSE or DMSO from the L1 stage did not differ from that of worms grown in medium alone. Exposure to SSE (1%) at the L4 larval stage had no effect on the progeny numbers in comparison to the DMSO control, revealing no direct effects of SSE on reproduction. The total load of congeners that were measured in SSE (Table 1) was still lower than that required for individual PCBs or PAH to reduce reproduction of *C. elegans* by approximately 50% in previous studies [25], [26]. Nevertheless, additive and synergistic effects might have been expected to occur given that several different xenobiotic compounds can induce the same isoform of CYP genes [27], [28]. The lack of a more obvious effect of SSE on reproduction in the present study could reflect a masking effect by the solvent and future work will use lower DMSO concentrations.

Interestingly, DMSO itself (1%) had a significant inhibitory effect on growth and development of *C. elegans* exposed from the L1 stage as compared to the medium control and inferred from reduced bioluminescence, smaller length, later progeny production and reduced progeny numbers at 94–98 h. The reduction in length was small, ca 5% or less after 49–71.5 h. By 74 h, the length of DMSO and medium control worms ceased to be significantly different, and the presence of L1 progeny in the medium control is likely to have contributed to the differences in bioluminescence. This was confirmed with the sterile strain PE328 where differences between treatments were much smaller. 1% DMSO is generally considered a safe vehicle concentration but it has also been reported that 1% was the lowest concentration with a significant effect of growth and development [29]. An effect of DMSO on progeny numbers was also observed when added at the L4 stage (approximately 30% reduction relative to medium). Others (using K-medium) reported a less than 10% reduction in reproduction for 1% DMSO [30].

The reduction in *C. elegans* longevity following SSE exposure from the L4 stage was observed only in the presence of the higher concentrations of the anti-metabolite drug, FUdR (400–600 µM). FUdR is routinely used at concentrations as high as 200–600 µM to prevent progeny production in lifespan assays [31], [32]. Depending on the stage of development of the worms when FUdR is added, it can prevent egg laying, hatching or development of small larvae. FUdR inhibits DNA and RNA synthesis, thus killing mitotic cells and inhibiting protein synthesis [33]. Our results are consistent with previous observations of increased mean nematode lifespan with FUdR exposure, particularly in liquid culture [34], [35]. This has also been noted more recently with genetic mutants [36]. One interpretation of the effect of SSE exposure is that it does not allow lifespan extension by FUdR to take place, i.e. the capacity for increase in lifespan is compromised in the presence of SSE. The potential mechanisms of action of FUdR have not been elucidated, but the reproductive system is known to influence the lifespan of *C. elegans* and removal of germline precursors by ablation (but not the whole gonad) triggers a longevity pathway, which is thought to involve steroid signalling [37], [38]. It would be of interest to determine whether FUdR induces similar signalling pathways and subsequently whether SSE interferes with this induction. The observations of the present study indicate that SSE exposure impacts on physiological mechanisms underlying lifespan which, if shown to be evolutionarily conserved, may have wider ecological and health related implications.

In conclusion, over and above vehicle effects, SSE caused a transient reduction in the energy status of *C. elegans*, delayed development and prevented lifespan extension by high FUdR concentrations. Our unique *C. elegans* biosensor responded with good sensitivity to concentrations of SSE that caused these effects. This biosensor reports on general metabolic impact, and while it is not diagnostic of the particular stress encountered, its sensitivity, rapidity and physiological relevance provides a powerful first line of assessment of effects of potentially–toxic, low-level, organic chemical mixtures, as demonstrated here with SSE.

The model system could contribute to the development of tiered toxicity testing of environmental chemical cocktails and to a reduction in the use of higher animals in testing and research. Since *C. elegans* is representative of a diverse and functionally important group of animals and carries many genes and pathways evolutionarily conserved in humans, this is not only relevant to the assessment of the ecological impact of chemical mixtures on natural ecosystems, but also to the understanding of mechanisms involved in potentially adverse effects on human health.

## Materials and Methods

### *Caenorhabditis elegans* Strains and Culture Conditions

Nematodes were maintained at 20°C on Nematode Growth Medium (NGM) agar plates seeded with *Escherichia coli* OP50 [39]. Strains PE254 and PE255 were used for the purpose of bioluminescence measurements. They are similar in that they contain the same *luc* transgene integrated in different chromosomes in the two strains [15]. In addition the ts sterile strain PE328 was also used (a derivative of PE254, combining the *luc* transgene with the *glp-4(bn2)* genetic background). *C. elegans* was grown in liquid medium by washing the nematodes from one NGM plate into 30 ml S-complete medium [40] plus *E. coli* OP50 (30 g/l) and incubating with shaking (160 rpm, 20°C). Nematode cultures were synchronised by bleaching gravid hermaphrodites and overnight hatching of eggs in M9 buffer [41].

### Sewage Sludge Extract Preparation

Sewage sludge pellets (2.025 kg) were ground and extracted with 1 litre of dichloromethane (DCM, Rathburn Chemicals, UK) at 55°C for 2 h; this was filtered (Whatman filter paper No 6) and dried by rotary evaporation; the resulting material was reconstituted in 400 ml dimethylsulphoxide (DMSO, 99.9% glass distilled grade Rathburn Chemicals, UK). The filtrate, referred to as sewage sludge extract (SSE), was stored in the dark, at room temperature. Concentrations of selected pollutants in SSE were determined by gas chromatography linked to mass spectrometry, as described in [42]. The same batch of SSE was used throughout the study.

### Experimental Design–ensuring Even Nematode Numbers between Experimental Conditions

Nematode numbers in liquid culture were adjusted to approximately 1.0×10^3^ /ml, except where otherwise stated. The range of numbers between liquid cultures used to set up different experiments was: 9.4 (±1.01)×10^2^ to 1.0 (±0.11)×10^3^ /ml for PE254 strain; 9.9 (±0.43)×10^2^ to 1.1 (±0.06)×10^3^ /ml for PE255; and 9.2 (±1.04)×10^2^ to 1.1 (±0.12)×10^3^ /ml for PE328. This was achieved by counting a minimum of 6×10 µl samples (up to 16×10 µl) from the shaken liquid culture (160 rpm), under a microscope. When aliquoting worms to 96 well plates, 12 ml of a shaken worm culture was carefully pipetted into a dispensing-trough, and kept at 160 rpm on a levelled shaking platform. Aliquots were taken from the mid-point of the liquid in the trough using a multi-pipettor. The dead volume was no less than 4.5 ml. Dispensing the worms in the presence of the bacteria, provided as food, prevented them from sticking to the plastic tips.

Due to inherent variability in worm numbers from well to well, we took the average measurement from a minimum of 5 wells (more usually 8 but as high as 12) as one single technical replicate. In our experience, differences in worm numbers between groups comprising this many wells are not statistically significant (please refer to Figure S1, providing counts and statistical data for worm numbers from different experiments with PE328). A minimum of 3 such technical replicates were included in experiments and experiments were repeated at different times.

### Bioluminescence Measurements to Determine Effect of Exposure to SSE on Metabolic Status

Synchronised cultures of PE254, PE255 and PE328 (approximately 2×10^3^ /ml) in 30 ml S-complete plus OP50 (30 g/l) were grown in 250 ml Erlenmeyer flasks (20°C, 160 rpm). At 46–47 h post-hatch, cultures were diluted to 1×10^3^ /ml in same medium and 25 µl aliquots transferred to 96 well plates (see ‘Experimental design’ above). For Figures 1A, 1B (PE254 and PE255), 25 µl of the serial dilutions of SSE in DMSO (Calbiochem, 100% purity) were then added resulting in final SSE concentrations of 0, 0.1, 0.25, 0.5 and 1.0% (final DMSO concentration 1%). Triplicate 96 well plates per experiment were incubated with shaking (160 rpm) at 20°C, ca 70% humidity.

For 24 h time courses (Figs. 1C, 1E), each strain PE254, PE255 and PE328, was exposed to a final SSE concentration of 0.5% (final DMSO concentration 1%). Strains PE254 and PE255 were tested within the same experiment and 4 independent experiments were carried out at different times (Fig. 1C), however, the period between 8 and 12 h exposure was only covered in one experiment and 14 h exposure in 2 experiments. Two sets of 3 independent experiments were carried out for strain PE328 (Fig. 1E): one set where exposure started at the L4 stage (35–36 h post-hatch at 25°C), the other at the adult stage (46–47 h at 25°C). Plates were incubated as above except for PE328, incubated at 25°C, and separate plates read for bioluminescence at timepoints over a 24 h period.

Experiments in which bioluminescence was measured during development (Fig. 2), involved exposure from the L1 stage (strain PE254, Fig. 2A; or PE328, Fig. 2C) or the L4 stage (46–47 h, PE254, Fig. 2B) to 1% SSE, 1% DMSO alone or S-complete. Although nematode numbers differed between the different PE254 experiments (range from 29.4±3.0 to 8.8±0.7 per well), there were no differences in nematode numbers between the treatments within any one experiment (p>0.05). Numbers for the PE328 experiments were similar between experiments (range from 23.0±2.6 to 26.3±1.9 per well). Separate plates were measured at each timepoint. Given that at 25°C nematodes develop 1.3 times faster [41], timepoints for the PE328 experiment were selected to be largely comparable to those of the PE254 strain at 20°C. Bioluminescence was read in a Clarity Luminometer (BioTek, Winooski, VT, USA) for 1 s, after injection of, and 3 minute incubation with, 25 µl of 3X concentrated luminescence buffer (S-complete plus 1% DMSO, 0.05% Triton X-100, and 100 µM D-luciferin), based on method described in [16].

### Progeny Number and Length Measurements

- **Progeny number.** This was assessed in 4 experiments with PE254 carried out independently on different days, by direct microscopic counting of hatched progeny following exposure of nematodes to SSE (1%) or DMSO (1%), from the L1 (post-hatch) or the L4 (47 h) larval stages. In two experiments, progeny number was determined from wells from 96 well plates set up for bioluminescence determination as described above. In the other 2 experiments, set up for determination of progeny number only, nematodes were placed in 6 well plates, 1 ml per well, 2 wells per treatment, and incubated with shaking at 20°C, ca 70% humidity. Numbers of hatched progeny were counted at 94–98 h post-hatch and expressed per adult nematode.
- **Length determination.** Sodium azide anesthetised nematodes (10 mM) were photographed using a Hamamatsu ORCA-ER digital camera linked to a Zeiss microscope equipped with a 2.5 Xs objective and the number of pixels converted to mm using the software Openlab 5 (Improvision®).

### *In vitro* Bioluminescence Measurements with Purified Luciferase

The ATP bioluminescence CLSII kit (Roche) was used according to manufacturer’s instructions. Final concentrations of SSE of 0.1, 0.25, 0.5 and 1% were tested, along with 1% DMSO and medium control (S-complete). ATP levels were kept constant at 1 mM final concentration. Bioluminescence signal was read in a Clarity luminometer and integrated for 10 s after automatic injection of the purified luciferase protein to the test samples. Three independent experiments were performed at different times, including 4 technical replicates of each treatment.

### Measurement of Fluorescence

Fluorescence was quantified in a Fluoroskan Ascent® (Labsystems) with the Ascent Software 2.6 (Thermo Scientific), using 485 excitation and 520 nm emission filters. Background measurements were subtracted from readings. Nematodes were set up in transparent Nunc Nunclon™ 96 well plates concomitantly to the set up for bioluminescence measurements. Fluorescence readings were carried out at the same timepoints as bioluminescence, but in contrast the same plates were measured repeatedly over time (set up in duplicate for two of the experiments and singly for the third). 8 wells were considered one technical replicate.

### Egg Laying Rate and Number of Eggs *in utero*

L1 larval stage worms **(**synchronised cultures of strain PE254) were exposed to SSE (1%) or DMSO (1%) on NGM plates. At L4 larval stage, worms were transferred to an intermediate plate of same composition. After a further 20 h, worms were actively egg laying and at least 8 worms were transferred to individual plates of the same composition [43]. The number of eggs released during each of the first two hours after transfer were determined. The overall rate of egg laying was calculated using the mean of these values. To determine the number of eggs carried in the uterus, 1 day old adult worms (n = 11–23) from NGM plates or from liquid cultures were individually transferred to 10 µl sodium hypochlorite (1%)/KOH (0.125 M) on a glass slide. This treatment dissolved the body of the adult animal and released the eggs, which were counted immediately.

### Lifespan Determination

Synchronised cultures of PE254 were grown as described for bioluminescence measurements. After 45.4 h the worm culture was diluted with S-complete plus OP50 (30 g/l) to 1×10^3^ /ml and 5-fluoro-2′-deoxyuridine (FUdR, Sigma-Aldrich) added to prevent progeny production (at 100, 400 or 600 µM final concentrations depending on the experiment) [31], [33]. This was followed by addition of SSE (1%) or DMSO (1%). Incubation was carried out at 20°C with shaking (160 rpm) in 125 ml conical glass flasks containing a total volume of 12 ml. Samples (100 µl) were taken at regular timepoints over the lifespan of the nematodes to assess numbers of live (movement when touched) and dead (unresponsive to touch) worms (ca 100 worms in each sample), by microscopic observation. Flasks were coded and all scoring was conducted blinded to treatment. In order to test the effects of SSE in the absence of FUdR, a temperature sensitive mutant [*glp-4(bn2)*; strain PE328 [15]] was used which does not reproduce at 25°C. This strain was grown at 15°C and shifted to the restrictive temperature (25°C) at the L4 larval stage and treated as described above for lifespan assays, except for the exclusion of FUdR and the incubation temperature (25°C).

### Data Analysis

Bioluminescence results were expressed as a percentage of the values obtained for control conditions (solvent alone). Error bars on figures represent standard error of mean (SEM). Effects of treatment on bioluminescence, purified luciferase activity, nematode length, progeny number, egg laying rate, and number of eggs *in utero* were compared using analysis of variance post hoc by the Holm-Sidak method unless stated otherwise. Where appropriate, a logarithmic or square root transformation was applied to data to comply with the assumptions of normality and homogeneity of variance. The analyses were conducted using SigmaPlot 11.0 and Minitab 15. The 5% level was adopted as a threshold for statistical significance.

Two-way ANOVAs were applied to bioluminescence data, with treatment and experiment as the two factors (Fig. 1A). Each experiment within Figure 1B was analysed separately, with treatment and replicate plate as the 2 factors. A significant effect of plate (p<0.001) and interaction between plate and treatment (p = 0.034) was only observed for the closed squares experiment. Two separate One-way ANOVAs were applied to summary data (Fig. 1D, short and long exposure). Repeated measures One-way ANOVA was applied to fluorescence data (Fig. 1E). The worms were measured (length) destructively at each timepoint (independent samples) and statistical analysis of each timepoint was carried out separately (Table 2). Homogeneity of variances could not be met for most timepoints except for 69 h (Box-cox transformed data to achieve normality, analysed with One-Way Anova) and One-Way Analysis of Variance on Ranks (Kruskal-Wallis) was applied to the other length data, followed by post hoc by the Dunn’s Method. Progeny number in Figure 4A was tested by One-way Analysis of variance followed by post hoc Dunnett testing which compared each treatment against the other 2, all differences significant (p<0.001).

Statistical analysis of lifespan data was carried out using GenStat 13. Logistic regression models using a binomial distribution and logit link function were used to model the proportions of nematodes surviving over time with respect to treatments (medium control, DMSO, SSE). Survival rates were estimated from % of sampled nematodes that were alive at each timepoint; this estimation assumes that all nematodes are available for sampling at each timepoint (no decrease in total numbers was observed over the course of the experiments). Cubic logistic models were used for data sets obtained in the presence of FUdR as they were found to provide an improved fit to the data over linear logistic model, whilst a linear logistic model was found to be adequate for the PE328 strain (sterile at 25°C). Overdispersion parameters were used when fitting the models to allow for the counts showing greater variability than would be expected from the binomial distribution. Effects of time, treatments and interaction of treatments with time were assessed using these models and the median lifespan (time when 50% of nematodes were alive) determined. Separate analyses were carried out for the data for 100, 400, 600 µM FUdR or no FUdR (PE328 strain) as these were tested in independent experiments.

Confidence limits for the median lifespan values were determined using bootstrap resampling. Bootstrap resamples of numbers alive for any given sampling time were generated by sampling from binomial distributions with probabilities set to the observed proportion surviving at that time. Median lifespan values were determined from repeatedly re-running the models using bootstrap resamples, rather than the observed data. In this way, bootstrap distributions of median lifespan values were built up and lower and upper limits of a 95% confidence interval were derived from these by taking the 2.5 and the 97.5 percentiles of the bootstrap distribution of median lifespan values.

## Supporting Information

Figure S1 **Worm numbers per well in replicate independent experiments with the PE328 strain.** Average number of worms per 96-well plate column (n = 8 wells; equivalent to one experimental technical replicate) taken from 2 independent PE328 experiments at the 4 h exposure timepoint (Figure 1E). After the luminescence readings, nematodes were pipetted out of the well (75 µl) and placed on a microscopic slide for counting. An extra 75 µl of S complete plus 0.01% tween was then added to the well and the first tip/pipette used to remove any remaining nematodes from the well for counting. Prior to discarding the tip, it was checked under the microscope for any worms adhering to it (0.01% tween minimises this) and counts were added together to obtain the total count for the well. In this example columns 1, 3, 5 were exposed to 1% DMSO control (average ± SEM: 30.0±1.8, experiment 1; 29±0.8, experiment 2) and columns 2, 4 and 6 to 0.5% SSE (30.2±1.3, experiment 1; 28.2±1.1, experiment 2). The minimum and maximum values observed in a well were: 18, 45 (experiment 1) and 17, 45 (experiment 2). No significant differences in nematode numbers were found between the 6 columns in each experiment (comprising 8 wells each): P>0.05 in experiment 1 and 2. Reducing the number of replicate wells within each column to 5 for the purpose of statistical testing had no effect and differences in worm numbers remained nonsignificant in both experiments (P>0.05). One way ANOVA applied to squared root transformed data.
(TIF)
