# Supplementary figures and images for: Impact of Sublethal Levels of Environmental Pollutants Found in Sewage Sludge on a Novel Caenorhabditis elegans Model Biosensor
Source: PLoS One. 2012 Oct 3;7(10):e46503. doi: 10.1371/journal.pone.0046503 (PMC3463613; doi:10.1371/journal.pone.0046503)

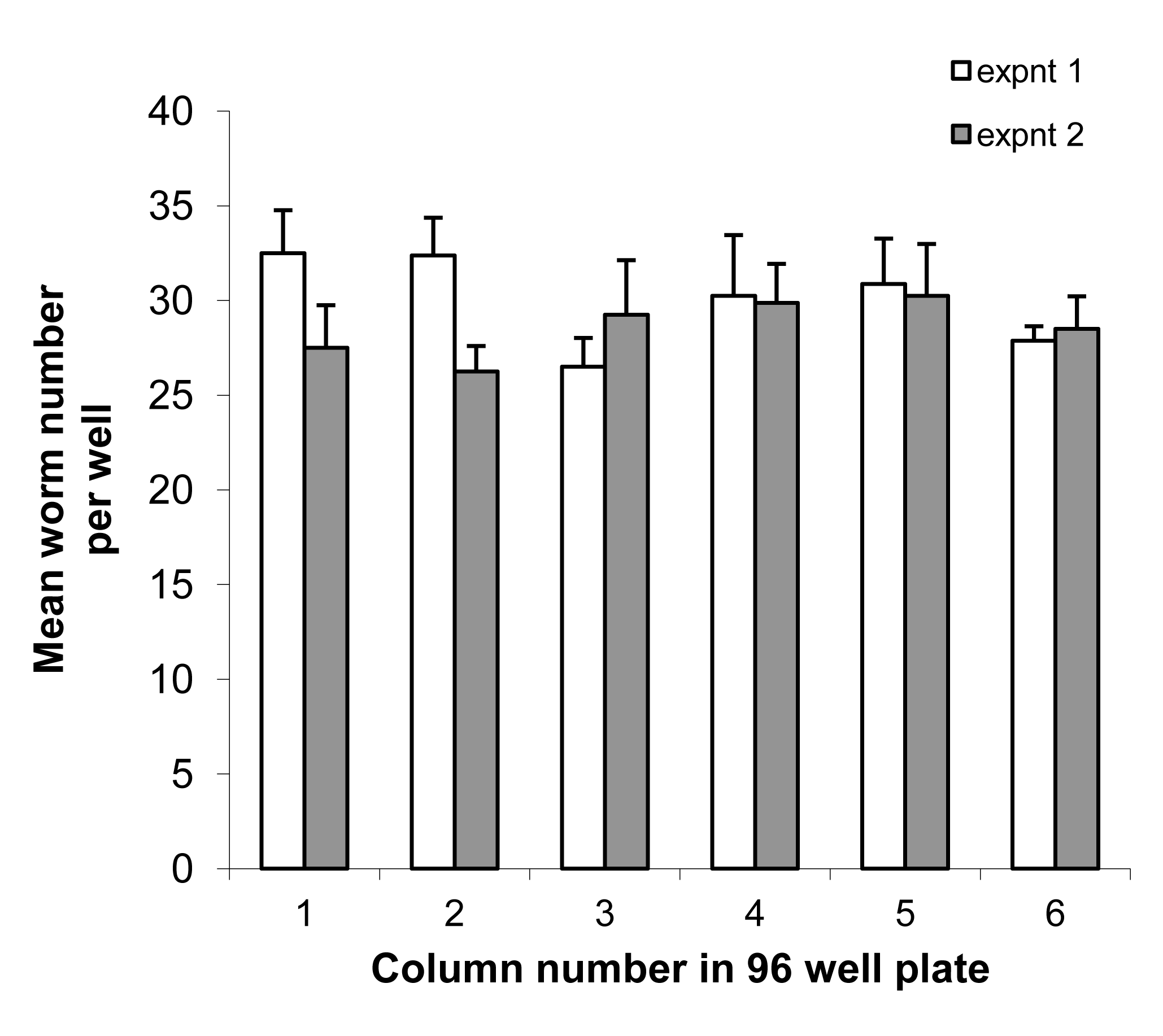

Supplement: Figure S1 — Worm numbers per well in replicate independent experiments with the PE328 strain. Average number of worms per 96-well plate column (n = 8 wells; equivalent to one experimental technical replicate) taken from 2 independent PE328 experiments at the 4 h exposure timepoint (Figure 1E). After the luminescence readings, nematodes were pipetted out of the well (75 µl) and placed on a microscopic slide for counting. An extra 75 µl of S complete plus 0.01% tween was then added to the well and the first tip/pipette used to remove any remaining nematodes from the well for counting. Prior to discarding the tip, it was checked under the microscope for any worms adhering to it (0.01% tween minimises this) and counts were added together to obtain the total count for the well. In this example columns 1, 3, 5 were exposed to 1% DMSO control (average ± SEM: 30.0±1.8, experiment 1; 29±0.8, experiment 2) and columns 2, 4 and 6 to 0.5% SSE (30.2±1.3, experiment 1; 28.2±1.1, experiment 2). The minimum and maximum values observed in a well were: 18, 45 (experiment 1) and 17, 45 (experiment 2). No significant differences in nematode numbers were found between the 6 columns in each experiment (comprising 8 wells each): P>0.05 in experiment 1 and 2. Reducing the number of replicate wells within each column to 5 for the purpose of statistical testing had no effect and differences in worm numbers remained nonsignificant in both experiments (P>0.05). One way ANOVA applied to squared root transformed data. (TIF) [file pone.0046503.s001.tif]
